# Oncoplastic and Reconstructive Breast Surgery: A Comprehensive Review

**DOI:** 10.7759/cureus.21763

**Published:** 2022-01-31

**Authors:** Bulent Citgez, Banu Yigit, Soysal Bas

**Affiliations:** 1 General Surgery, Uskudar University Faculty of Medicine, Memorial Hospital, Istanbul, TUR; 2 General Surgery, Elazig Fethi Sekin City Hospital, Elazig, TUR; 3 Plastic, Reconstructive and Aesthetic Surgery, University of Health Sciences, Sisli Hamidiye Etfal Teaching and Research Hospital, Istanbul, TUR

**Keywords:** surgical margin, reconstruction, radiotherapy, incision, breast-conserving surgery, oncoplastic breast surgery

## Abstract

Most of the approaches that were valid until recently in breast cancer surgery have undergone significant changes with rising awareness, increasing number of patients, and knowledge. It is important to repair the damage caused by surgical treatment performed in accordance with oncological principles and to obtain good cosmetic results. The quality-of-life indexes increase and body image is positively affected by the development of oncoplastic surgery and reconstruction techniques.

The oncoplastic techniques are commonly used for the closure of glandular defects. Surgeons must pay attention to the breast volume, tumor location, the amount of breast tissue that would be removed, and the oncoplastic technique that may be required. Oncoplastic breast surgery allows wide local excision of the mass with good cosmetic results. In addition, a contralateral breast lift, breast augmentation or breast reduction may be required to accommodate the conceptually reconstructed breast. The use of oncoplastic breast surgery techniques results in lower mastectomy rates with equivalent local and long-term survival rates as compared with mastectomy and offers women the option of plastic and reconstructive interventions performed at the time of initial surgery. Mastectomy may be needed for large tumors, as breast-conserving surgery may not be possible or may not produce satisfactory cosmetic results. Breast reconstruction methods after mastectomy include autologous or implant-based breast reconstructions, which can be performed at the same time as the breast cancer surgery (immediate reconstruction) or at a later time (delayed reconstruction).

Oncoplastic and reconstructive breast surgery minimizes the impact of breast cancer surgery and yields equivalent survival outcomes without psychological morbidity. With advanced techniques, better breast image than before can also be achieved. In this review, the technical details of oncoplastic breast surgery, surgical margin positivity management, reconstruction methods, radiation therapy given after reconstruction surgery, radiologic imaging modalities, and management of complications are discussed.

## Introduction and background

Halsted's radical mastectomy has been transferred to less extensive procedures defined as breast-conserving surgery (BCS), which is based on the principles of complete resection of the primary tumor with negative margins. The success of radiotherapy (RT) plays an important role in these improvements. Longer survival rates after treatment for breast cancer have been directly influenced by a better understanding of the molecular bases of breast cancer biology and the further development of adjuvant therapies. For these patients, the quality-of-life expectancy and the preservation of breast cosmesis are increasingly important in the treatment of breast cancer. Breast-conserving surgery, which was brought into routine practice with the initiatives of Veronesi et al. and Fisher et al., has evolved into the concept of 'oncoplastic surgery' in parallel with the demands and expectations of the patients [1,2].

Oncoplastic breast surgery (OBS) includes techniques created by combining the principles of surgical oncology with plastic and reconstructive surgery to provide acceptable aesthetic results in the treatment of breast cancer. The essential differences between OBS and conventional BCS are that OBS can provide larger volumes of excised tissue, less required re-excision rates, and reduced rates of positive margins [3]. The main goals of OBS are complete excision of the tumor, obtaining negative surgical margins, achieving excellent cosmetic outcomes, and if possible oncological surgery with concurrent reconstruction. Oncoplastic surgery and reconstruction techniques with implant-based or autologous tissues are used if the standard BCS operation with safe surgical margins is either impossible or creates a large deformity in the breast. Other indications include the patient’s desire, tumor characteristics, size and shape of the breast, and surgeon's experience [4-6].

## Review

Oncoplastic approaches to breast conservation

Oncoplastic breast surgery techniques are generally classified according to the breast size, location of the expected tumor resection, and the ratio of breast volume to resection volume. They are divided into two levels i.e., level-1 and level-2. Glandular density, patient-related risk factors (smoking, obesity, diabetes, previous surgery, etc.), adjuvant treatments, patient preferences and expectations are other specific factors that will influence decision making with regards to the OBS technique [7,8].

Level-1 oncoplastic techniques include basic segmental mastectomy techniques that require excision of less than 20% of the breast volume in small to moderate-sized breasts with minimal ptosis. The objective is tumor excision without skin resection, and to repair partial breast defects effectively to reduce the risk of deformity that will occur with the effect of RT. The main principles are the localization of the tumor, planning the incision, dissecting the subcutaneous flap widely beyond the tumor, identifying the tumor, removing the specimen, undermining the breast anterior to the pectoralis fascia, marking the tumor cavity, and closure of the defect with mobilized tissue pillars [8].

Level-2 techniques are applied in cases that require excision of 20% to 50% of the breast volume and are often accompanied by skin excision. The strength of this technique lies in obtaining a negative margin by allowing large amounts of breast tissue to be resected, and preventing radiation toxicity, especially in women with large breast sizes. Breast cancer-related lymphedema may occur in patients with macromastia, especially after axillary surgery and RT, with inadequate lymphatic drainage and limited effect of gravity on the lymphatics. Reduction mammoplasty, which prevents or reduces breast lymphedema after RT is an option for patients with macromastia. One of the greatest contributions of level-2 techniques to BCS is that they are often safe to resect multicentric tumors. Thus, tumor beds that are brought together with the safe margin obtained by resection and reduction, can be converted into a single boost area that RT can be safely applied [9].

Oncoplastic breast surgery technique selection according to tumor localization

**Figure 1:**
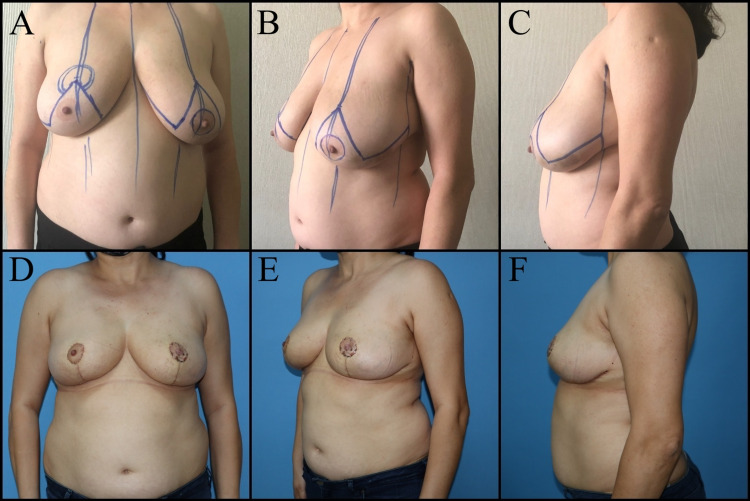
Forty-six-year-old female patient has left retro areolar ductal carcinoma in-situ. Right breast reduction mammoplasty, left breast wise pattern mastopexy and NAC reconstruction with graft was planned. A, B, C: Preoperative view. D, E, F: Postoperative one-month views. NAC: Nipple-areolar complex

Oncoplastic procedures are defined based on which quadrant the tumor is located: Batwing mastopexy lumpectomy for central (periareolar) tumors, racquet mammoplasty for upper outer quadrant tumors, round block/donut mastopexy (Benelli's technique) for upper quadrant tumors, reduction mammoplasty/lumpectomy for lower quadrant tumors (Figure 1) [10].

Yang et al. classified the techniques according to the distance from the nipple-areolar complex (NAC). Batwing mastopexy is applied to lesions located within 4 cm from the center of the nipple in the upper middle quadrant. Racquet mammoplasty is performed for lesions located within 6 cm to 8 cm from the center of the nipple in the upper outer quadrant. A rotational flap is applied for lesions located in the upper inner quadrant. Parallelogram mastopexy is recommended for tumors located more than 8 cm from the nipple in the upper middle quadrant. Although Yang et al. recommended round block mastopexy for tumors in the upper outer quadrant, we now know it can be applied to tumors in all quadrants of the breast. The V mammoplasty, J mammoplasty and reduction mammoplasty/lumpectomy methods have been recommended for tumors located in the lower quadrants [11].

Managing positive margins after oncoplastic surgery

Tumor size, histological type, extensive intraductal component, high grade, human epidermal growth factor receptor 2 (HER2) positivity, lymph node positivity, dense breast tissue, obesity, microcalcifications on mammography (MMG), and multifocality are significant risk factors affecting the positive surgical margin in breast cancer [12]. Intraoperative diagnostic methods, such as frozen section analysis, cavity shaving, intraoperative ultrasound (US), and specimen magnetomyography (MMG) can be used to determine the positive surgical margin [13].

The meta-analyses by Losken et al. showed that the rate of positive margin in OBS was less than that of conventional BCS [14]. In the presence of positive surgical margins in OBS, mastectomy was predominantly performed on these patients in the past. However, re-excision of margins or secondary mammoplasty currently offers an alternative to mastectomy [15]. On the other hand, patients undergoing reverse T, J, or V mammoplasty have an increased risk of skin necrosis after a mastectomy incision, as the incisions are close to the inframammary fold (IMF) in the lower pole of the breast [16].

There is still a higher incidence of mastectomy in OBS than in BCS in the presence of a positive surgical margin. The reasons for the more common use of mastectomy in positive surgical margins after OBS are that re-excision is technically more difficult, wide resection in a case of re-excision results in small breast size and asymmetrical breasts can pose a serious aesthetic problem. It is also difficult to localize the initial tumor bed after breast reshaping. Thus, it is important to have a clip placed in the tumor bed cavity and to select the appropriate mammoplasty technique [15].

Reconstruction

Breast reconstruction can be performed at the same time as the mastectomy (immediate reconstruction) or can be delayed until after adjuvant treatments (delayed reconstruction). Both immediate and delayed reconstruction can be performed using autologous tissues, implants or a combination of both. Autologous tissue-based reconstruction can be performed with pedicled or free flaps (musculocutaneous or muscle-sparing). Implant-based reconstruction can be done in one-stage (direct-to-implant) or two-stage (tissue expander followed by a permanent implant). The one-stage option has been improved by the diffusion of synthetic meshes and acellular dermal matrices (ADMs), which can be used to enlarge the sub-muscular pocket or to cover the implant above the muscle. The implant could be placed under or over the pectoralis major muscle [17, 18].

Immediate Reconstruction

The breast reconstruction is done simultaneously with mastectomy. The advantages of immediate reconstruction are the reduced number of operations, lower costs, single-stage recovery and consequently shorter hospital stay, positive mood and higher self-esteem due to positive body image. The most important disadvantage is skin necrosis, which may result from circulation problems and possible complications associated with the flap in the setting of mastectomy skin necrosis [19].

Delayed Reconstruction

**Figure 2:**
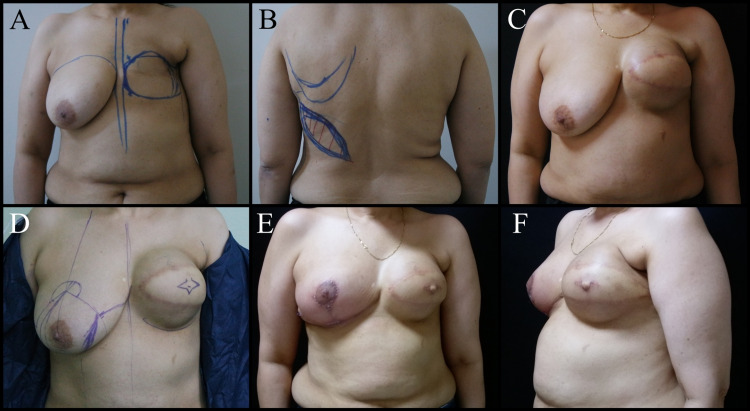
Fifty-seven-year-old female patient. A mastectomy was performed on the left breast three years ago due to invasive ductal carcinoma. A, B: Breast reconstruction plan with latissimus dorsi muscle skin flap and expander. C: Post-expanded view of the left breast. D: Reduction mammoplasty to the right breast, NAC reconstruction plan to the left breast. E and F: Postoperative one-month views. NAC: Nipple-areola complex

**Figure 3:**
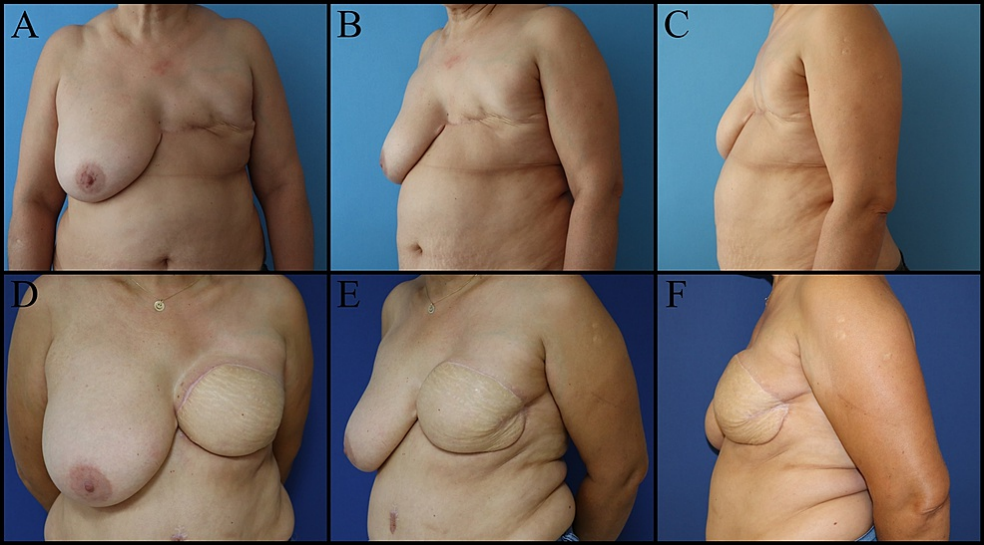
Forty-five-year-old female patient. Mastectomy was performed on the left breast seven years ago for invasive ductal carcinoma. Breast reconstruction with a DIEP flap was planned for the left breast. A, B, C: Preoperative views. D, E, F: Postoperative one-year views. DIEP: Deep inferior epigastric perforators

Breast reconstruction can be performed at any time following mastectomy. Thus, the hematological effects of chemotherapy improve and the skin changes become stable after RT. Its major advantage is that it offers the opportunity to plan the second stage surgery per the regression of the skin changes after RT. The disadvantages are the loss of anatomical features of the breast and skin sheath, increased number of operations and higher costs (Figure 2, 3) [17,20].

Incision choice and technical challenges of immediate breast reconstruction

The decision for therapeutic mastectomy depends on the size and location of the tumor, tumor size relative to breast size, the amount of skin excised, and the degree of ptosis. Mastectomies are classified into three types namely, skin-sparing mastectomy (SSM), skin reduction mastectomy (SRM) and nipple-sparing mastectomy (NSM), which allow immediate reconstruction and require selection of specific incision. Skin-sparing mastectomy aims for the complete removal of the breast tissue, areola and nipple while preserving the skin of the breast. Nipple-sparing mastectomy includes resection of the entire breast parenchyma while preserving the dermis and epidermis of the NAC. Nipple-sparing mastectomy is a safe technique in patients with small/medium breast size and low-grade ptosis without NAC invasion. Skin reduction mastectomy is applied to remove excess skin in patients with large or ptotic breasts. Surgeons should be wary of predisposing factors, including a history of RT, obesity, high breast volume, diabetes, and smoking, for immediate reconstruction complications [21].

There are some points to consider when deciding on the incision type. Skin incisions should be drawn with the patient in the upright sitting or standing position. The midline is determined from the sternal notch to the umbilicus. The IMF and lateral fold must be identified. Length, width and projection of the contralateral breast should be measured. Incisions applied to IMF are inconspicuous and can be hidden below the breast. Axillary interventions should be carried out through the incisions for mastectomy. The incision scar, surrounding skin texture, the position of the NAC and IMF are valid tools to assess aesthetic outcomes after reconstructive surgery [22].

Skin-sparing mastectomy is classified by the amount of tissue to be removed. Type I SSM is a type of mastectomy performed prophylactically or in patients with small breasts and no ptosis. Only the NAC is removed. Type II SSM involves removing nipple-areola, skin overlying superficial tumors and previous biopsy incision. Type III SSM is used when the tumor or previous biopsy scar is remote from the areola, and separate incisions are performed. Type IV SSM is used when contralateral breast reduction is planned for large and ptotic breasts. Wise-pattern (WP) incisions involving areola are preferred. The incisions used in SSM can be modified according to the size of the breast, reconstruction type, and areola diameter [23].

**Figure 4:**
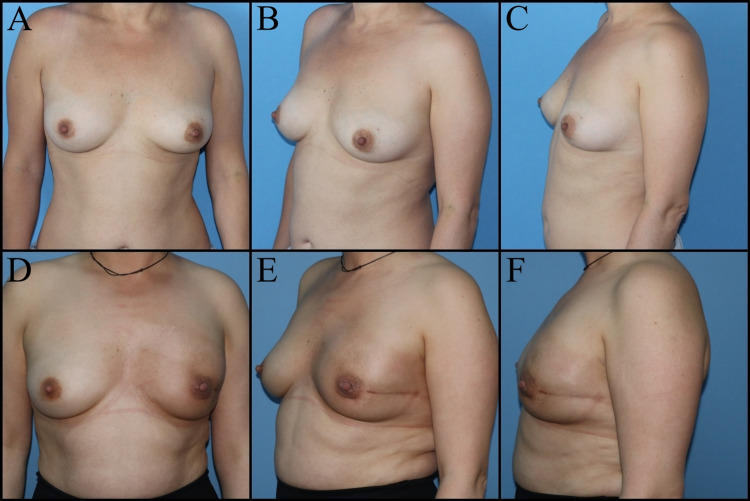
Thirty-eight-year-old female patient planned for subcutaneous mastectomy and immediate implant-based reconstruction due to ductal carcinoma in situ of the left breast. A, B, C: Preoperative images. D, E, F: Postoperative eighth-month views.

The most commonly used skin incisions for NSM include the lateral radial, and IMF incisions (Figure 4).

It is difficult to access the upper quadrants in medium- to large-sized breasts via the IMF approach. Periareolar incisions involving more than 30% of the areolar circumference are an independent risk factor for NAC necrosis and loss. A study by Park et al. compared the lateral radial and IMF incisions for NSM. They found that there is a tendency to select IMF incisions in patients with lower breast volume and that permanent implant applications are mostly made through this incision. Periareolar incisions were found to be more prominent for autologous reconstruction [24]. However, periareolar incisions appear to be more prone to complications such as NAC ischemia and necrosis that may require revision surgery [24, 25].

Studies investigating factors affecting patients’ satisfaction have revealed that radial incisions extending to the areola are less satisfactory in terms of aesthetic outcomes, loss of nipple sensitivity, and numbness compared to IMF incisions. Nipple projection and position can be displaced by radial incision scar. Patients with excess breast tissue removed, high body mass index (BMI), and large implants are less satisfied with their breast shape [26].

The main challenge of immediate breast reconstruction is inadequate space due to the short incision line. Excision is performed in circular directions to increase exposure during surgery. Skin flaps should be gently retracted. While preparing mastectomy flaps, the parasternal line on the medial boundary and the latissimus dorsi muscle on the lateral boundary should not be exceeded. Areas of nonviable skin should be intraoperatively detected and excised. The skin flaps should be elevated superficially to the enveloping fascia of the breast, and care should be taken not to make the flaps too thin or too thick. Breast tissue is more adherent to the skin in the lower quadrants, and the subcutaneous tissue is thicker in the upper outer quadrant. The breast skin receives its blood supply from the subdermal plexus, and avulsing the skin and subcutaneous tissue during NSM may also damage the subdermal plexus [27].

We can consider tumescent mastectomy in well-selected patients. The skin flaps are infiltrated using a tumescent solution, prepared by mixing Ringer’s lactate plus 1% lidocaine plus 1:1000 epinephrine. It also facilitates surgical dissection and reduces bleeding. The tumescent technique does not increase the risk of flap necrosis, wound infection, and wound dehiscence. Intraoperative perfusion tests can predict and prevent complications [28].

The NAC malposition may occur after implant-based reconstruction. Small breast implants may require the removal of excess skin in patients with large ptotic breasts. Incisions close to the edge of the pectoralis major muscle tolerate pressure better. On the other hand, incisions close to the lateral and inferior borders of the breast have a greater pressure load and cause delayed wound healing. Leaving a 2 mm to 3 mm rim of tissue around the nipple bundle after removing the duct bundle preserves its blood supply [29].

**Figure 5:**
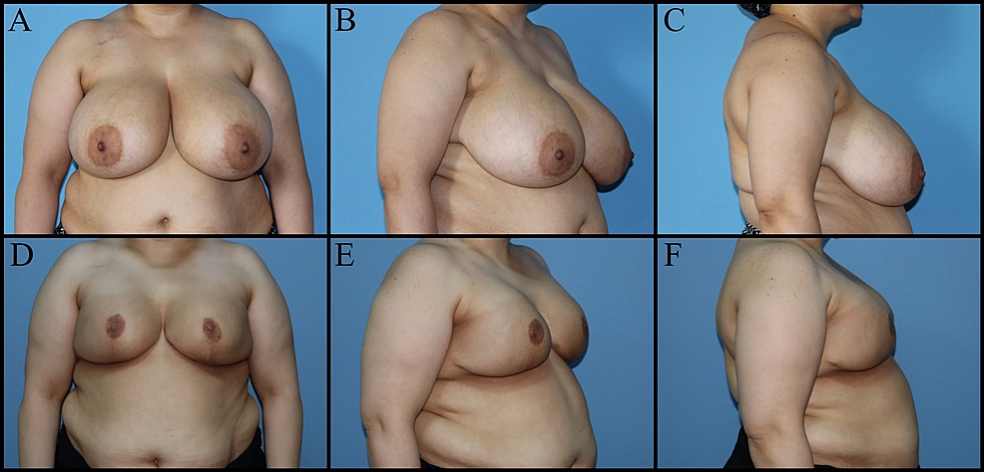
Fifty-one-year-old female patient with family history of breast cancer and BRCA gene positivity. The patient underwent a bilateral subcutaneous mastectomy, wise pattern skin reduction, dermal flap, and implant-based immediate breast reconstruction. A, B, C: Preoperative views. D, E, F: Postoperative views. BRCA gene: Breast cancer gene

Dermal barrier flaps contribute to wound healing by increasing blood flow at the suture line and form a secondary barrier that protects the implant in case of wound dehiscence [30]. Wise pattern incision, inferior dermal flap, and immediate implant use in medium and large breasts is another safe reconstruction option. The package created by attaching the dermal flap and pectoral muscle is fully vascularized. In addition, they do not carry the disadvantages of acellular dermal matrices (Figure 5) [31].

Nipple-sparing mastectomy is technically more difficult to perform in patients with previous breast surgery scars. Scar tissue does not need to be removed unless it causes nutritional problems or compromises oncological safety. If the previous scar is planned to be revised, it would be better to postpone it to another session [27].

Radiotherapy after breast reconstruction

Factors associated with the choice of breast reconstruction method after mastectomy include oncological treatment type, additional treatments, patient-related factors, surgeon's experience and patient's preference. There are studies comparing the efficacy of RT between immediate and delayed reconstruction. On the other hand, no randomized controlled trials have been conducted to compare the efficacy of RT between autologous and implant-based breast reconstruction. Patients who will receive postoperative RT can be determined in advance with the use of advanced diagnostic methods. It is important to plan a patient-specific, multidisciplinary treatment and to explain the possible side effects of RT [32].

Immediate breast reconstruction with implant eliminates negative psychological effects of mastectomy and appears to be a lower cost in the short term. However, this procedure is associated with a higher risk of capsular contracture after RT and an increased risk of implant loss in those with axillary lymph node dissection. In addition, there is a higher risk of complications compared to reconstruction with autologous tissue. The advantages of two-stage tissue-expander breast reconstruction include removal of irradiated tissue, correction of breast asymmetry, and better cosmetic outcomes in patients receiving RT. On the other hand, complication rates accounted for most cases undergoing two-stage reconstruction are higher than one-stage reconstruction with autologous tissue or implants [33]. Autologous breast reconstruction is more expensive than a comparative tissue expander, but it is deemed to be cost-effective with many savings derived from a one-stage operation. In patients undergoing RT, autologous tissue reconstruction is associated with fewer complications, better cosmetic results, reduced skin-sensation loss, and higher quality of life scores than reconstruction with a tissue expander or implant. This procedure also requires a highly experienced team in the field of reconstructive surgery and donor site morbidity should be minimized [32].

Radiation therapy can alter NAC vitality and lead to ischemia, partial/total loss, areolar sufferance, nipple projection loss, hypopigmentation, and asymmetry. It is recommended to evaluate perfusion of NAC during surgery, not to resect tissue aggressively, and to prefer lateral-radial incisions [34].

Factors that increase the risk of implant loss include tissue expander not covered by acellular dermal matrix (ADM) or serratus muscle, thin skin envelope, early expander-to-implant exchange (< 3 months), IMF incisions, and axillary dissection [35]. Prepectoral placement of an implant requires appropriate skin thickness, wider subpectoral pocket and use of prepectoral ADM. The surgeon also does not need to cut through or separate the muscles. No animation deformity or displacement of the implant is seen with activity. Target volume should be carefully planned for patients with positive or 'close' margins [36]. Prepectoral implant irradiation has been shown to be safe with similar complication rates as subpectoral implant irradiation [37,38]. Multiple studies have demonstrated increased rates of capsular contracture with subpectoral implant placement. This is because RT causes fibrosis, tissue thickening, malposition, and compresses the implant surrounded by the muscle [39].

The old techniques of RT, utilization of high energy photons, and chest wall boost also adversely affect the results of breast reconstruction. It is possible to deliver the desired dose distribution and establish dose constraints for healthy organs at risk with the development of machines used in RT [40]. The combination of deep inspiration breath-hold (DIBH) and volumetric-modulated arc therapy (VMAT) results in significantly lower doses to the heart, lungs, and contralateral breast [41]. The risk of infection, skin necrosis and implant loss is increased in patients who have undergone breast reconstruction, for whom we need to apply additional radiation doses, such as surgical margin positivity [42].

To date, there is no level-1 evidence on the optimal cut-off value of interval from surgery to RT for breast cancer because surgical techniques, RT and risk factors that can change side effects have not been evaluated homogeneously [43]. Appropriate timing of postoperative RT, RT fractionation schemes, and long-term oncologic outcomes are being evaluated in multiple ongoing phase-3 clinical trials. Patients receiving RT should be monitored for tissue edema, infection, and skin fibrosis. Moisturizing creams, non-steroidal anti-inflammatory drugs (NSAIDs), antibiotics, arm and shoulder exercises, scar massage, physiotherapy, compression bra and pectoxylin/vitamin E supplementation may be recommended [44,45].

Radiology

Imaging methods are helpful in the selection of surgical incision and technique in the preoperative period, tumor localization and margin evaluation in the intraoperative period, and in determining the need for reoperation or mastectomy after lumpectomy in the postoperative period.

Breast Imaging-Reporting and Data System (BI-RADS) is used to classify breast density on MMG into four categories from A to D. The high proportion of glandular tissue and stroma in category C or D breast density makes it difficult to detect breast cancer. Therefore, MMG has a higher sensitivity in detecting breast cancer in the evaluation of breasts consisting predominantly of adipose tissue (80% to 98%) compared to very dense breasts (30% to 48%) [46]. On the other hand, fat necrosis is commonly seen in category A or B breast density when we apply level-1 oncoplastic techniques that require mammary tissue to be separated from skin flaps and resected along with the pectoralis major fascia.

The most widely accepted and used intraoperative imaging technique to determine the incision or dissection plan of OBS is ultrasonography (US). The intraoperative US is helpful in the detection of non-palpable masses, real-time dynamic imaging, radiological confirmation of negative margins, less amount of tissue removal, and cavity evaluation. Its disadvantages are that it is operator dependent, requires experience, and is not determinative in microcalcifications and multifocal small tumors. In the randomized controlled trial of COBALT, intraoperative US guidance for breast cancer excision was found to be superior to palpation-guided excision in obtaining an intact surgical margin [47]. In addition, other studies on margin management in BCS have also proven that the intraoperative US is superior to other methods in obtaining adequate surgical margins with sufficient experience [48,49].

The aims of close follow-up after OBS are the early diagnosis of recurrent disease, reduction of requested biopsies, and decreased frequency of false-positive findings due to the misleading effects of postoperative changes. Ultrasound imaging of the breast may also help radiologists evaluate some lesions that are difficult to see on a mammogram, especially in edema secondary to treatment. A mammogram should be obtained six to 12 months after treatment ends. After that, a follow-up mammogram at least once a year is sufficient. In some cases, dense breast tissue and scarring may be associated with an increased risk of misdiagnosis, and MRI can help determine whether the change is normal maturation of the scar or a recurrence of cancer. Although not currently recommended for routine screening, breast MRI may be useful in patients with invasive lobular cancer or extremely dense breast tissue, in patients receiving neoadjuvant therapy or, in the presence of conflicting findings on MMG and US [50].

The presence of early postoperative complications, such as hematoma, seroma, fat necrosis, and edema may manifest as palpable or mammographically detected masses. Late complications may also include fat necrosis, scar formation, retraction, dystrophic calcifications, and tissue asymmetry. The development of a new mass, microcalcifications, architectural distortion or an area of increased density at the scar site, a new or enlarging area of asymmetry during follow-up, should be suggestive of breast cancer recurrence. Mammographic spot compression views can be applied with or without magnification. If previous examination results are available, the findings should be compared with prior mammograms [51].

Management of complications

Complications of OBS are related to oncological excision of the breast, axillary surgery and subsequent reconstruction surgery. Major complications in OBS are caused by a lack of respect for pedicles and blood flow. Familial predisposition, genetic risk factors, comorbidities, physical status (American Society of Anesthesiology score), BMI, smoking, physical activity, lifestyle, musculoskeletal disorders, patient preferences and desires, local and systemic impairment concerning disease burden are patient-related factors [52-54]. Toxicities related to chemotherapy and RT should also be considered in the management of complications.

Seroma

It is a common complication arising from the donor site of autologous breast reconstruction, breast, and axilla. The most important risk factors for seroma are age, obesity and the amount of breast tissue removed. Management options for the treatment of recurrent seromas include observation for spontaneous resolution, percutaneous aspiration, compression, seroma-desis and/or sclerotherapy [55].

Wound Infection

Surgical wound infection is a result of ischemia, compromised vasculature and necrosis of the skin flap. Signs of wound infection include pain, swelling, warmth or redness, cloudy fluid or pus discharge. Necrotic tissue and slough should be debrided and wound culture must be taken after debridement. Antibiotic therapy should be combined with incision and drainage of abscesses. *Staphylococcus aureus*, along with *Staphylococcus epidermidis* are considered to be major causative agents. Antibiotic therapy should be further modified according to culture and susceptibility results [55].

Skin Flap Necrosis

Wound closure technique, tight sutures, hematoma, radiation, smoking, additional comorbidities such as obesity and diabetes are risk factors. Topical antibiotics, such as mupirocin and fusidic acid, are used in the treatment of necrosis. If flap adherence and demarcation occur, appropriate and early debridement accelerate wound healing [56].

Hematoma

Bleeding diathesis, hypertension, and inadequate hemostasis predispose to hematoma formation. Postoperative hematoma caused by excessive bleeding or failure of the evacuation of the blood can all be treated by reopening the wound. Reoperation is performed by evacuating the hematoma and performing hemostasis, followed by an aspirative drain [55].

Fat Necrosis

Although it is a common complication, radiological imaging often cannot clearly distinguish between recurrence and fat necrosis on follow-up. Therefore, if the diagnosis cannot be established, imaging-guided biopsy or excision should be performed [57].

Lymphedema

It is recognized as a common complication after axillary surgery or RT. It may cause limitations in the shoulder and arm movements. The swelling may also contribute to shoulder dysfunction and limited arm movements [55].

Necrosis of the Nipple-Areola Complex

**Figure 6:**
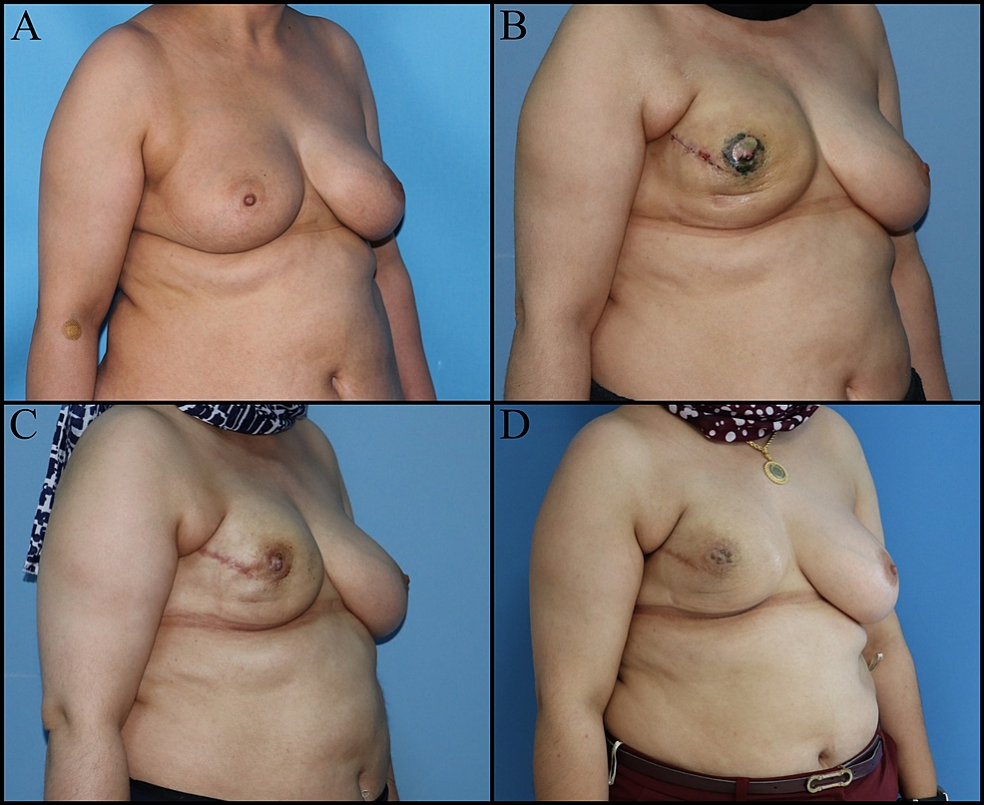
Thirty-nine-year-old female patient planned for NSM and immediate implant-based breast reconstruction due to right ductal carcinoma in situ. A: Preoperative view. B: Partial nipple necrosis, postoperative one-month view. C: Postoperative two months. D: Postoperative eight months, pigment loss in the nipple. NSM: Nipple-sparing mastectomy

Total or partial necrosis of the NAC may develop (Figure 6).

The loss of the NAC may occur for a variety of reasons: different types of flaps (long or thin and superficial pedicles, kinking or compression of the pedicle), oncological excision types complicating the flap, and patient-related causes such as smoking, diabetes, obesity, and hypertension. Pale nipple and absence of capillary circulation imply arterial insufficiency. Bruising and change in nipple color around a varicose vein is a sign of venous insufficiency. If nipple congestion occurs, treatment would be to relieve pressure by removing the sutures. Most treatment regimens still use aspirin, heparin or dextran for arterial insufficiency. Pulmonary edema is a potential complication of dextran, while aspirin and heparin can cause bleeding and hematoma. These medications should be used with caution. Hydrotherapy, local heparin injection and hyperbaric oxygen therapy are effective in venous congestion [58]. Reconstruction of the NAC can be planned following local infection control, serial surgical debridement, and wound care.

Loss of Nipple Sensation

The NAC is innervated by the anterior and lateral cutaneous branches of the second to the fifth intercostal nerves. Oncological resection and reconstruction techniques can damage these nerves. While nipple sensation sometimes returns within several weeks, some loss of feeling might be permanent [59].

Breast/Nipple Deformities & Asymmetry

Deformities may develop as a result of complications as well as due to technical reasons. Reconstruction surgery may be required to provide symmetry and correct the deformity.

Flap Necrosis Following Autologous Breast Reconstruction

Necrosis may occur as a result of circulation problems in flaps (parenchymal or dermoglandular, fasciocutaneous, muscle, free flaps) used for reconstruction. The skin island of the flap can be used for monitoring the circulation. If the flap doesn’t have a skin island, clinical follow-up should be performed. Local signs of infection such as odor, pain, redness, exudate or discharge indicate necrosis. After local infection control, facilitating wound drainage, debridement, removal of devitalized necrotic tissue and wound care, reconstruction is planned according to the size and type of the defect [60].

Flap Donor Site Morbidity

**Figure 7:**
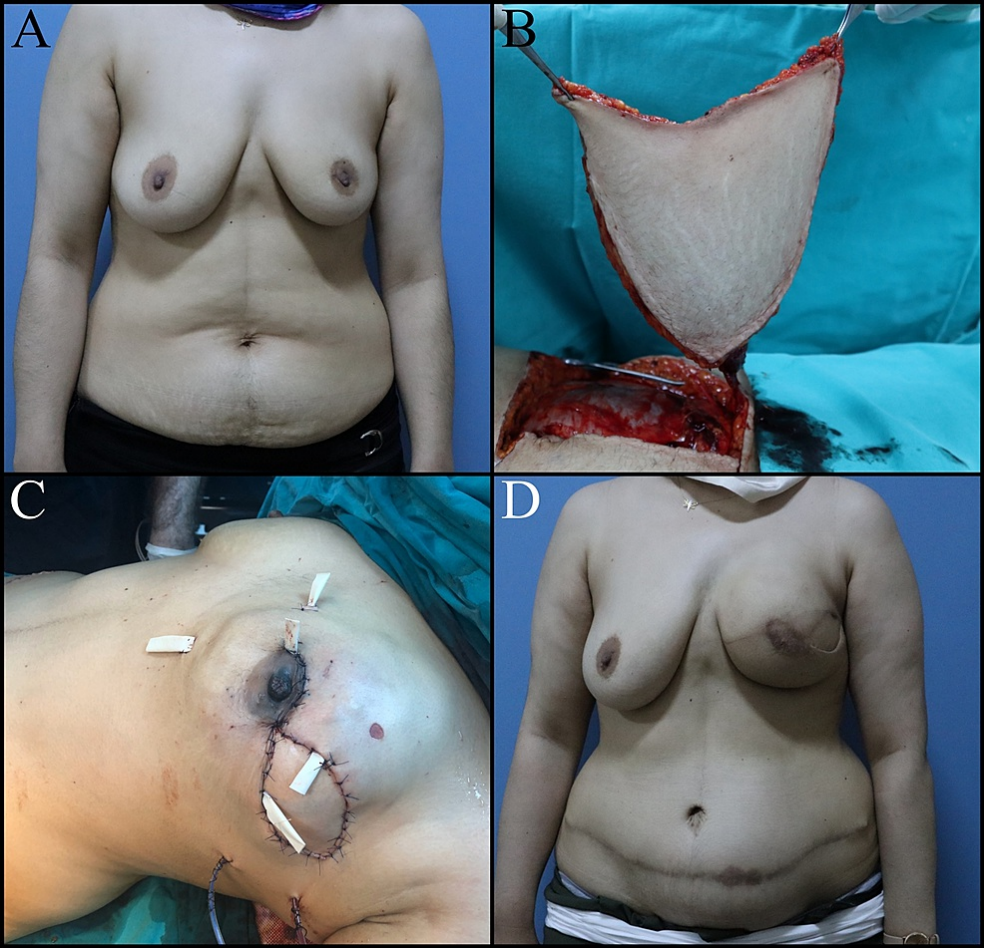
Forty-two-year-old female patient. The patient planned for left subcutaneous mastectomy and breast reconstruction with simultaneous DIEP flap due to invasive ductal carcinoma. A: Preoperative image. B: DIEP flap elevation. C: Immediate postoperative view. D: Abdominal hernia, postoperative first-year view. DIEP: Deep inferior epigastric perforators

Donor site complications are seen especially in free flaps or distant pedicle flaps. Latissimus dorsi flap necrosis significantly impacts shoulder movements and this is resolved by effective application of physiotherapy. Transverse rectus abdominis myocutaneous (TRAM) flap reconstruction carries the possibility of significant complications of donor site, including weakness of anterior abdominal wall muscles and abdominal hernia that may require surgical repair. These complications are extremely rare as no muscle is removed with the perforator flaps [DIEP and superficial inferior epigastric artery (SIEA)] (Figure 7) [61,62].

Breast implant complications

Implants used to restore the shape and volume of the breast may cause complications such as capsular contracture, necrosis, displacement, asymmetry, wound dehiscence, and infection. Smooth surface implants have a higher rate of capsular contracture. It is important to avoid implant pocket irrigation with betadine to reduce the risk of capsule contracture [63]. Treatment of the capsular contracture consists of capsulotomy, capsulectomy and replacement of the implant. Textured implants are associated with an increased risk of infection, and a lower risk of capsular contracture [64]. The possibility of infection should be considered in the presence of persistent swelling or pain around the breast implant. The patients are initially treated with antibiotics. If the infection cannot be controlled with antibiotics, the implant should be removed. Local complications such as displacement, asymmetry, wound dehiscence may require corrective or revision surgery. Serial debridement is performed in the presence of breast skin necrosis. The selection of the reconstructive method should depend on the defect characteristics. Autologous reconstruction is indicated when there is insufficient tissue on the chest wall to cover and support the breast implant [65]. Animation deformity can be observed in patients undergoing subpectoral implant-based breast reconstruction. It is usually corrected by a pocket change to the subcutaneous position or selective nerve ablation with botox injections [66].

## Conclusions

Oncoplastic and reconstructive breast surgery improves women’s quality of life and reduces the psychological distress associated with mastectomy. Surgeons should focus on oncological outcomes but also consider functional and aesthetic outcomes when planning resections. Failures of oncoplastic and reconstructive breast surgery often arise from a lack of judgement and planning. The risk of positive margins and the need to convert to mastectomy should always be considered, and the incisions and pedicles should be appropriately planned.
